# Seeking antiviral drugs to inhibit SARS-CoV-2 RNA dependent RNA polymerase: A molecular docking analysis

**DOI:** 10.1371/journal.pone.0268909

**Published:** 2022-05-31

**Authors:** Ibrahim Khater, Aaya Nassar

**Affiliations:** 1 Biophysics Department, Faculty of Science, Cairo University, Giza, Egypt; 2 Department of Clinical Research and Leadership, School of Medicine and Health Sciences, George Washington University, Washington, DC, United States of America; University of Akron, UNITED STATES

## Abstract

COVID-19 outbreak associated with the severe acute respiratory syndrome coronavirus (SARS-CoV-2) raised health concerns across the globe and has been considered highly transmissible between people. In attempts for finding therapeutic treatment for the new disease, this work has focused on examining the polymerase inhibitors against the SARS-CoV-2 nsp12 and co-factors nsp8 and nsp7. Several polymerase inhibitors were examined against PDB ID: 6M71 using computational analysis evaluating the ligand’s binding affinity to replicating groove to the active site. The findings of this analysis showed Cytarabine of -5.65 Kcal/mol with the highest binding probability (70%) to replicating groove of 6M71. The complex stability was then examined over 19 ns molecular dynamics simulation suggesting that Cytarabine might be possible potent inhibitor for the SARS-CoV-2 RNA Dependent RNA Polymerase.

## 1. Introduction

The 2019 coronavirus disease (COVID-19) pandemic associated with the severe acute respiratory syndrome coronavirus (SARS-CoV-2) has become a humanitarian crisis [1]. Similar epidemics associated with viral infections of the coronaviruses were reported over the past two decades including the pandemics of the previous severe acute respiratory syndrome coronavirus (SARS-CoV) identified in late year of 2002 [2–4] and the influenza pandemic (H1N1) identified in the year of 2009 [5, 6], in addition to the Middle East Respiratory Syndrome coronavirus (MERS-CoV) that was found in Saudi Arabia during the year of 2012 [7–10].

Human coronaviruses (HCoVs) target the human respiratory system, mainly the lungs. Past reported pandemics related to such coronavirus infection belonged to the *Alphacoronaviruses* family of 229E and NL63 strains, and the *Betacoronaviruses* family of OC43, HKU1, SARS, and MERS strains [11]. The most aggressive coronavirus infection was associated with both SARS and MERS strains. The newly emerged SARS-CoV-2 virus is highly contagious and transmissible across the nations, especially with the new variant surges. Analyzing the genomic sequence of the newly emerged SARS-CoV-2 demonstrated approximately sequence identity of 88% to that of SARS genomic sequence validating SARS-CoV-2 as a new member of *Betacoronaviruses* [11], [12–14]. Epidemiological studies reported the symptoms of the SARS-CoV-2 similar to those symptoms caused by other *Betacoronaviruses* [13, 15–18].

The HCoVs are described as lengthy positive single-stranded RNA viruses of about 30K bp [19] and carrying structural and non-structural proteins. There are four structural proteins that characterize all coronaviruses: the spike protein (S), the nucleocapsid protein (N), the membrane protein (M), and the envelope protein (E), as well as non-structural proteins like proteases (nsp3 and nsp5) and the RNA-dependent-RNA polymerase RdRp (nsp12) [20–23]. SARS-CoV-2 is a positive strand RNA virus with numerous component replication-and-transcription complexes of viral nonstructural proteins (nsps) that control its replication [24, 25]. Nsp12 function is dependent on accessory proteins like as nsp7 and nsp8 [22, 26]. Nsp12 contains N-terminal domain (NiRAN), an interface domain, and C-terminal RdRp domain [27]. Fingers, palm, and thumb subdomains make up the RNA dependent RNA polymerase (RdRp) domain, where nsp7 and nsp8 subunits attach to the thumb and an additional copy of nsp8 attach itself to the fingers [22, 26, 28].

The conserved polymerase motifs A-G in the palm domain form the active site of the RdRp domain. The traditional divalent-cation-binding residue D618, which is conserved in most viral polymerases, is found in motif A. In the turn between two β-strands, motif C contains the catalytic residues (759-SDD-761), these catalytic residues are also conserved in most viral RdRps, with the first residue being either serine or glycine. Motif D stabilizes the core structure while motif E controls the flexibility of the thumb. Motif F contains K545, K551 and R553 which are responsible for rNTP binding and positioning. Motif G predicted to be involved in positioning of template overhang [20, 23, 29–31].

Developing a therapeutic antiviral treatment that is safe and effective would take few years, therefore, in February 2020, the World Health Organization (WHO) research forum on the coronavirus disease 2019 recommended the evaluation of the commonly used approved antiviral regimens against COVID-19 [32, 33]. The regimen recommended searching all previously approved antiviral drugs against the coronavirus disease, this screening process would speed the process of finding a quick antiviral drug for COVID-19. Comprehensive computational studies repurposed approved antiviral drugs against SARS-CoV-2 [34–38], where commonly used approved antiviral drugs were examined against SARS-CoV-2 protein structures including the RNA-dependent-RNA polymerase [39–44], papin-like protease [45–48], and the main protease [49–54], using in-silico molecular docking to seek potential SARS-CoV-2 inhibitors by analyzing the binding probabilities [55–62]. The findings of those studies suggested that the top scoring drugs could be used as lead compounds for further experimental validation for the development of effective antiviral treatment against SARS-CoV-2.

A large-scale analysis of regularly used antiviral medications may provide therapeutic possibilities that may be positioned to speed up experimental and clinical testing. In this study, we looked through the drug library for authorized antiviral medications to investigate possible antiviral activity against SARS-CoV-2. The current research work is an in-silico analysis seeking authorized antiviral treatments that inhibit SARS-CoV-2 RNA-dependent-RNA polymerase (SARS-CoV-2 RdRp). We used an in-silico approach to shortlist polymerase inhibitor candidate drugs, and we analyzed published studies. Our analysis identified a few candidate drugs, some of which are already being investigated for COVID-19 treatment and can serve as a basis for prioritizing additional viable COVID-19 candidate drugs.

## 2. Materials and methods

### 2.1. SARS-CoV-2 RdRp Structure

The SARS-CoV-2 RdRp (nsp12) complexed with its cofactors nsp8 and nsp7 in apo form was obtained from the Protein Data Bank (PDB ID: 6M71). The 6M71 structure consists of four chains: the A chain (nsp12), the B and the D chains (nsp8) and the C chain (nsp7). Proteins were prepared for molecular docking analysis using the AutoDock Vina protocol [63].

### 2.2. Polymerase inhibitors optimization and molecular docking

The structures of the polymerase inhibitors were downloaded from the DrugBank [64]. The MMFF94 force field function of Avogadro software was used to optimize the geometry of all inhibitors [65]. Molecular Docking analysis was performed using the AutoDock Vina protocol [63]. The docking was performed against the entire protein to evaluate the free natural affinity of the binding ligand for the replicating groove without pushing the ligand to dock selectively to the active region. The docking was repeated 10 times for each ligand, and the affinity of docking was assessed using the docking scores and the likelihood of binding to the replicating groove.

### 2.3. Analysis of interactions between inhibitors and RdRp

The fully automated protein–ligand interaction profiler (PLIP) web tool was used. PLIP detects and visualizes protein–ligand interaction patterns in 3D structures, either directly from the PDB or from user-supplied structures [66]. Results are presented in 3D interaction diagrams for manual examination, either online using JSmol or offline using PyMOL, as well as XML and text files for additional processing for each binding site [66]. The PLIP web tool was used to examine the interactions established between the inhibitors and the SARS-CoV-2 RdRp to evaluate the docking results. All interactions are described, down to the atom level, allowing for detailed analysis of specific binding properties. Ligand efficiency is the binding affinity divided by a measure of the size of a ligand [67]. Compounds that can provide the desired binding affinity with fewer atoms are considered efficient [68–70].

### 2.4. Molecular dynamics simulations

CHARMM-GUI was used to create the protein topologies and the parameter files [71–73]. GROMACS-2019 software package [74] and CHARMM36 force field [75] were used for the molecular dynamics simulation. The system was solvated with TIP3P water in the add solvation box [76] and the entire complexes were neutralized by using the Monte-Carlo ion-placing approach to add appropriate amounts of K+ and Cl ions. The system was energy-minimized for 5000 steps using the steepest descent approach before simulations [77] and equilibrated for 125 ps at constant number of molecules, volume, and temperature (NVT). Finally, the molecular dynamics simulations were performed for 1900 ps (19 ns) at constant temperature (310 K), pressure (1 atm), and number of molecules (NPT ensemble), and was good enough for RMSD straight line [78], [79]. Ramachandran plot analysis was carried out for validating the docked complex structure. The root mean square deviation (RMSD) of the protein atom backbone, the radius of gyration (Rg) and the number of hydrogen bonds and solvent accessible surface area (SASA) were plotted as a function of time [80]. The average root mean square fluctuation (RMSF) was then plotted as a function of residues number. Compressed coordinates were measured every 10 ps (1900 frames).

## 3. Results and discussion

Molecular docking approach was employed on 6M71 and the mean values of the docking scores and the probabilities of binding to the replicating groove are shown in Table 1. Cytarabine (-5.65 Kcal/mol) had the best likelihood (70%) of binding to the replicating groove of 6M71 based on the docking results.

**Table 1:** List of the molecular docking scores in Kcal/mol calculated using AutoDock Vina against SARS-CoV-2 (PDB ID: 6M71, nsp12-nsp8-nsp7). The docking procedure was done ten times for each ligand, and the likelihood of binding to the replicating groove were determined. The highest probabilities of binding to PDB ID: 6M71 are shown in bold red color.

Polymerase Inhibitor Tested	PDB ID: 6M71	
	ΔG (Kcal/mol)	Probability of binding to replicating groove
Mithramycin	-8.72 ± 0.74	30%
2’-O-Methylcytidine	-5.58 ± 0.06	10%
Rifapentine	-8.23 ± 0.33	10%
Galidesivir	-6.65 ± 0.36	20%
Dactinomycin	-9.13 ± 0.71	20%
Aureothricin	-5.00 ± 0.14	0%
Thiolutin	-4.88 ± 0.33	0%
Cytarabine	**-5.65 ± 0.18**	**70%**
Juglone	-5.54 ± 0.13	0%
IDX-184	-6.79 ± 0.47	10%
Ribavirin	-6.28 ± 0.261	30%
sofosbuvir	-6.45 ± 0.28	30%
Resistomycin	-8.32 ± 0.24	0%
Deacetylcolchiceine	-6.58 ± 0.39	30%
Streptolydigin	-7.85 ± 0.47	50%
Avigan	-6.58 ± 0.29	30%
Remdesivir	-7.74 ± 0.28	10%

**Fig 1:**
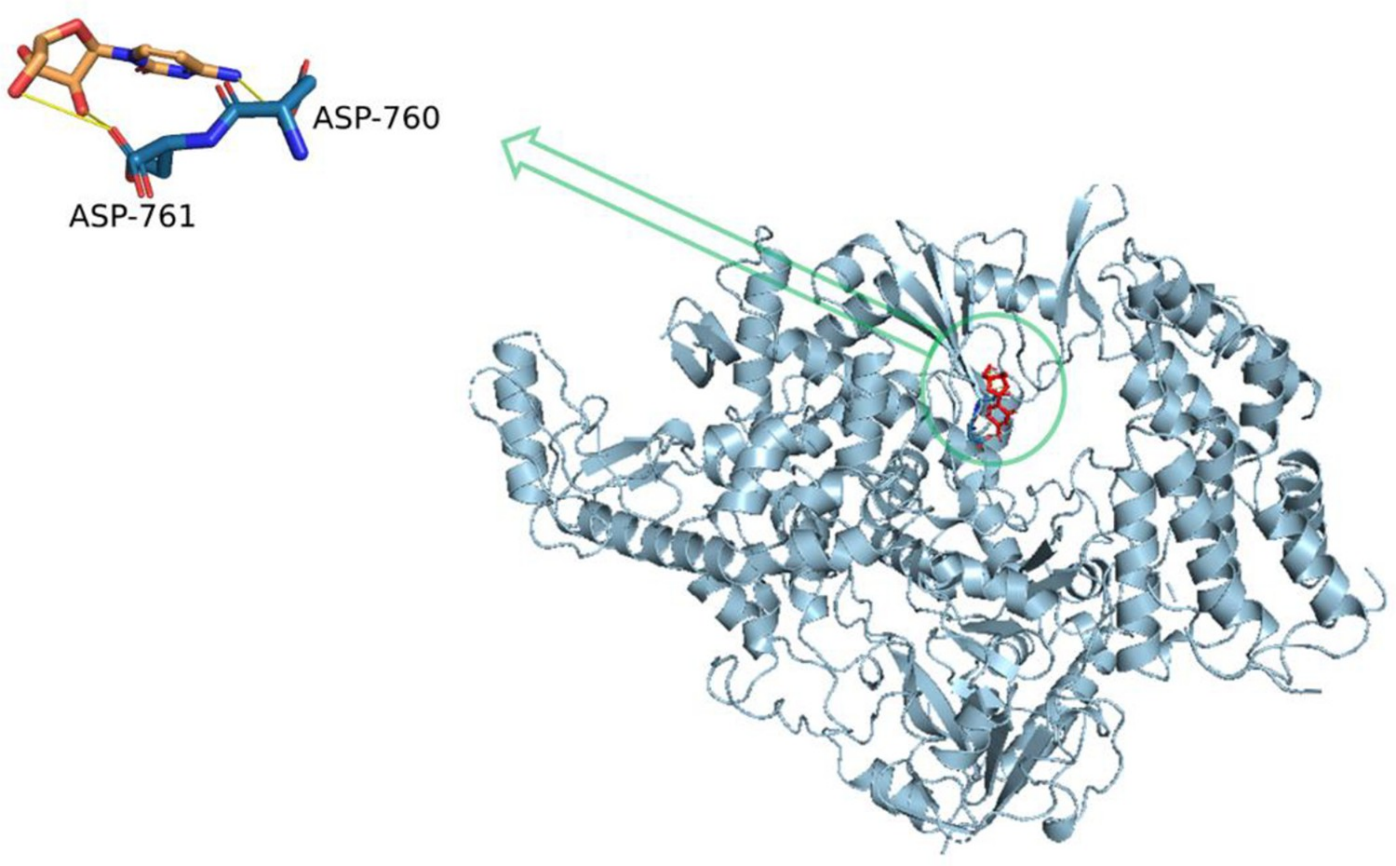
Cytarabine, with a -5.65 Kcal/mol, has the highest likelihood (70%) of binding to RdRp’s replicating groove (6M71). Ligands are labeled with a three-letter code, whereas protein residues are tagged with a licorice color. H-bonds are represented by solid yellow lines.

To investigate the likely reasons for the binding energy differences, we examined the formed complexes using PLIP web server. Ligands are shown in licorice color, while the protein residues are labeled with one-letter code. The H-bonds are represented in solid yellow lines. Fig 1 illustrates the formed interactions between Cytarabine and 6M71 after the molecular docking. The three H-bonds formed between Cytarabine molecules and 6m71, two of them formed with D761 and one was formed with D760 (catalytic residues in C motif). The catalytic residues’ function was limited by hydrogen bonds formed with the active site pocket, which prevented them from being shared in virus replication.

**Fig 2:**
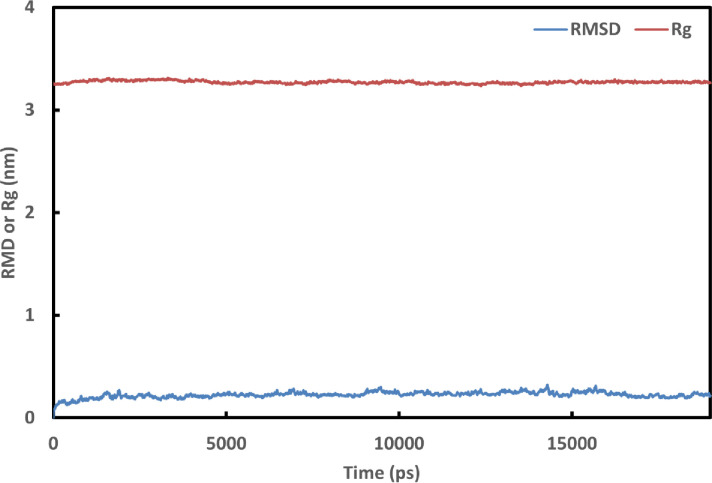
Graphs of RMSD for the backbone atoms and Rg as a function of time are shown over the course of a 19-ns simulation.

**Fig 3:**
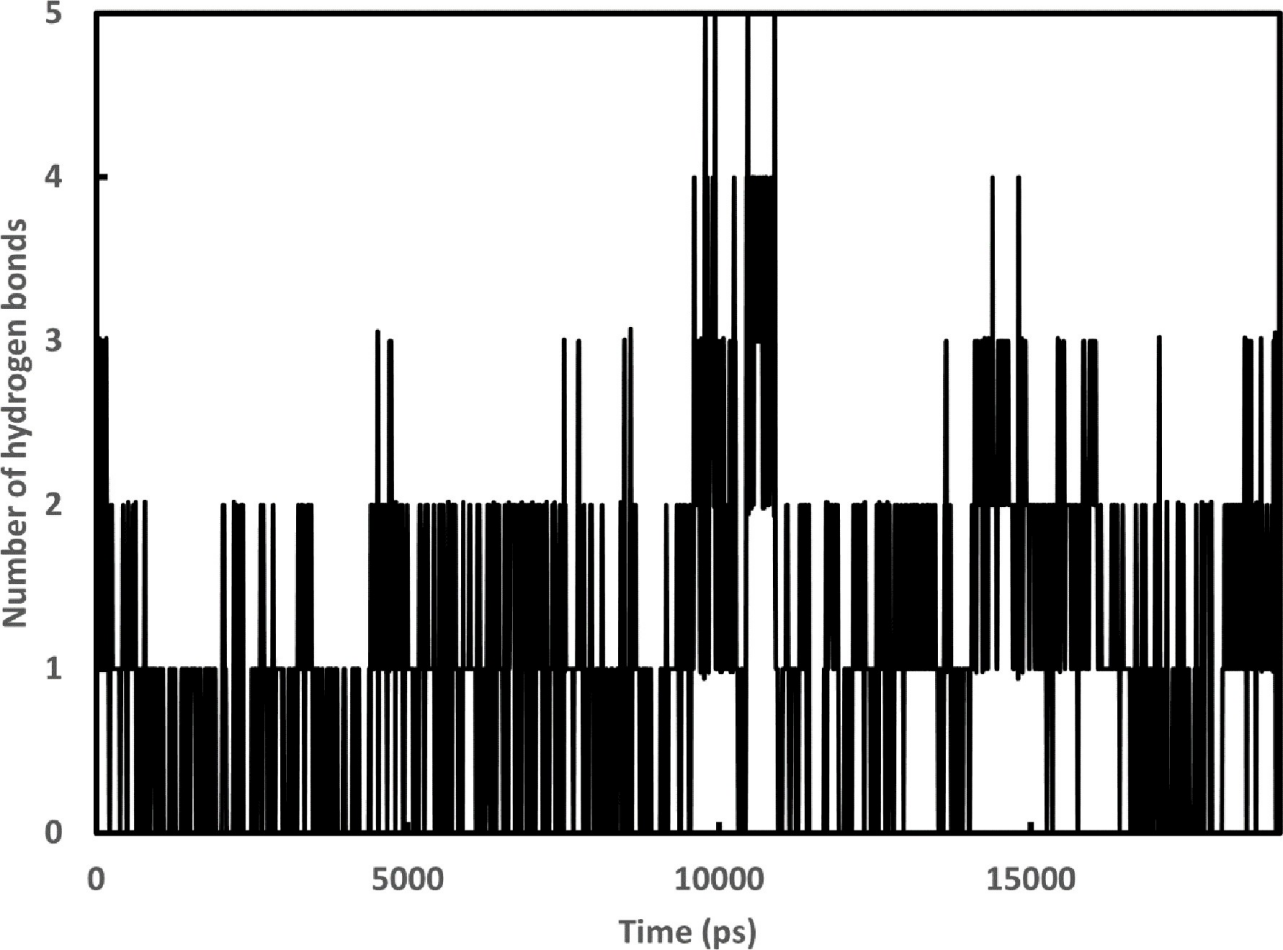
Graphs depicting the number of hydrogen bonds formed between RdRp and Cytarabine as a function of time during a 19-ns simulation.

**Fig 4:**
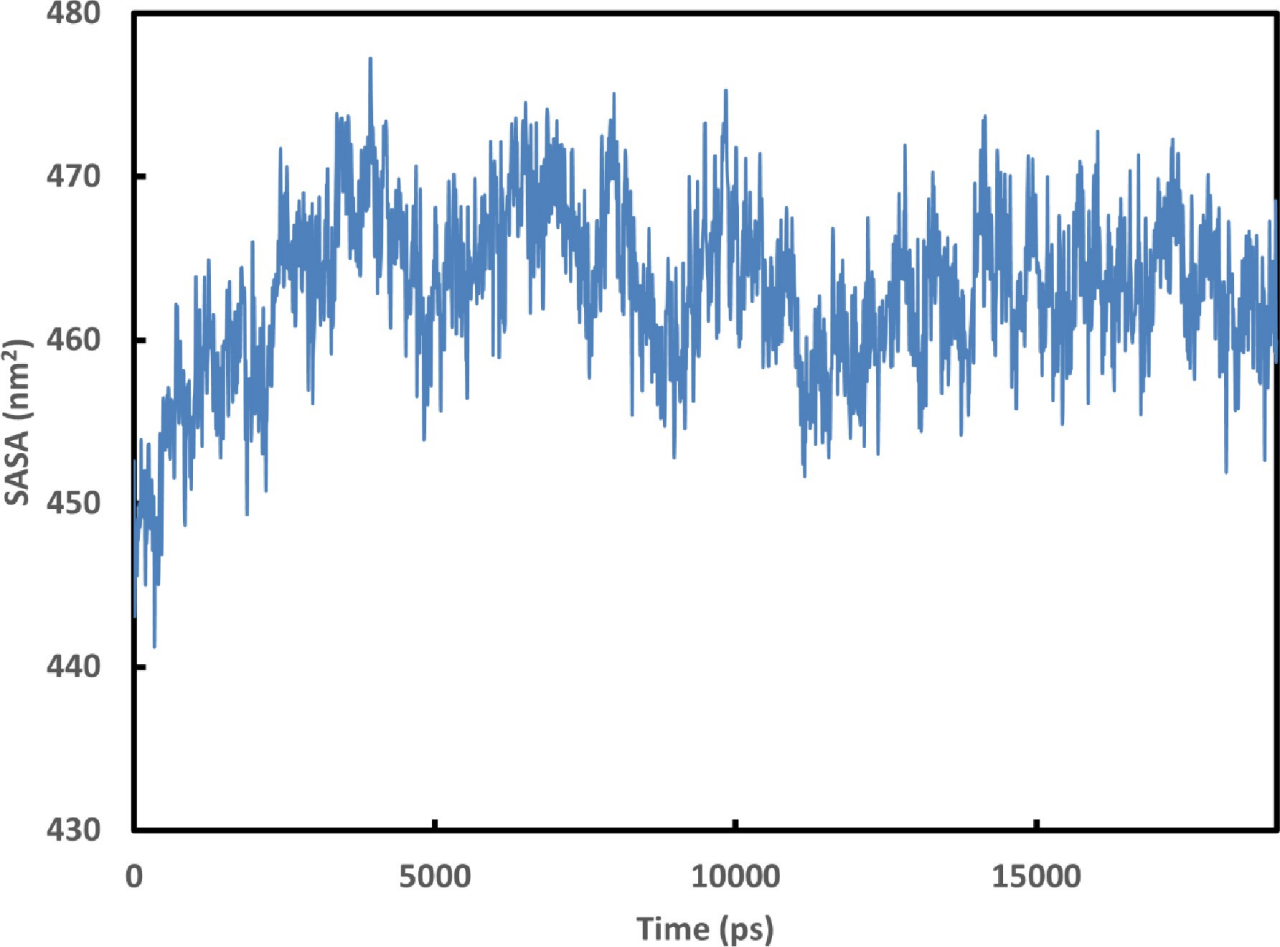
Graphs of SASA of RdRp as a function of time for a 19 ns simulation.

**Fig 5:**
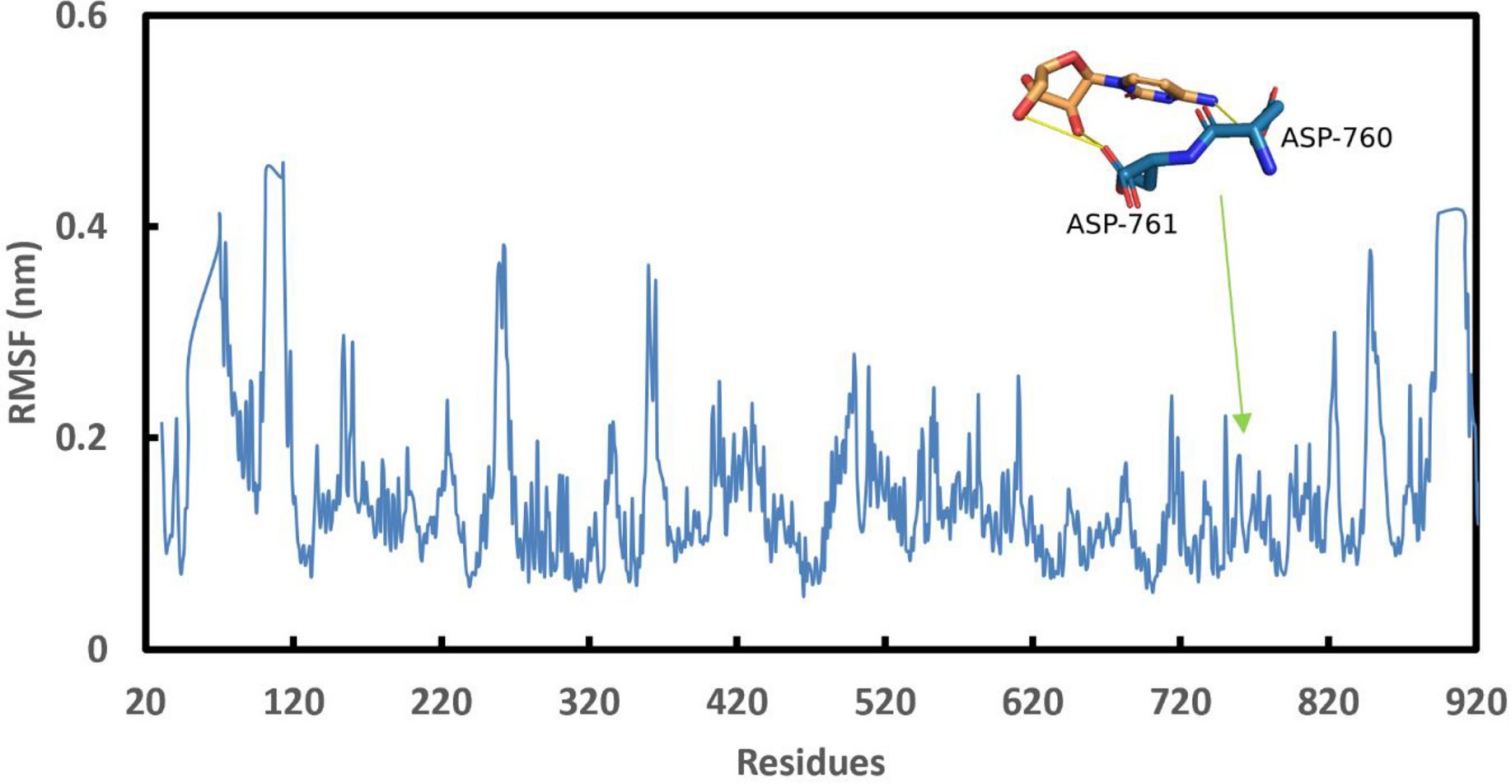
Graphs depicting the average RMSF per residue throughout the course of a 19-ns RdRp simulation.

The docking mechanism created fast connections with the legends and the protein, which could be unstable [81]. The molecular dynamics simulations provide information on the stability of the generated complexes’ molecular interactions. Based on the binding energy, Cytarabine expressed their highest probability to bind to the 6M71 protein. The stability of the complex was assessed using the RMSD for the backbone atoms of 6M71 protein in comparison to the initial structures [82]. Fig 2 shows a graph of the RMSD values of the 6M71-Cytarabine complex after 1900 ps stabilization (19 ns). Furthermore, the stability of the complex was assessed by graphing Rg [82]. Fig 2 shows the computed Rg values along the simulation time scale, showing that the parameter is stable for the 6M71-Cytarabine complex over time. Fig 3 depicts the number of hydrogen bonds that exist between 6M71 and Cytarabine. During the simulation, the number of hydrogen bonds in the complex varies from 0 to 5. Similar findings were made using SASA analysis, which represented the solvent-defined protein surface and its orientation during the folding process, resulting in changes in the exposed and buried areas of the protein surface area. Fig 4 depicts the results of SASA plotted over the simulation time. Fig 4 as well displays a convincing SASA value for the 6M71-Cytarabine solvation profile, indicating a stable structure and robust binding contact with the Cytarabine. Fig 5 shows the average RMSF for 6M71 over 19 ns per residue. The variations of the 6M71 catalytic residues ASP-60 and ASP-61, which form hydrogen bonds with Cytarabine, are less than 1.5 A^o^, indicating that the contact is robust and stable. In summary, the approved drugs (Cytarabine, Streptolydigin, Ribavirin, Sofosbuvir, Deacetylcolchiceine, Mithramycin, Avigan, Remdesivir, IDX-184) can bind to SARS-CoV-2 RdRp, with different binding energies.

## 4. Conclusion

Since the start of the COVID-19 pandemic, a large and impressive number of research studies and clinical trials have been launched attempting to find treatments for the rapidly spreading coronavirus infection. The prospective approach of medication repurposing research employing in-silico screening techniques has been shown to be successful in identifying active compounds against SARS-CoV-2-targeted proteins. The goal of this work was to find prospective candidates among the licensed antiviral medications that can bind and interact with SARS-CoV-2 RdRp using a drug repurposing approach. The study examined a variety of polymerase inhibitors that are currently on the market to inhibit the SARS-CoV-2 RNA-dependent-RNA polymerase. Because Cytarabine showed the highest likelihood of binding to the active site pocket of the SARS-CoV-2, the results of the current in-silico molecular docking analysis employing binding affinity and interactions may support the use of Cytarabine as a possible candidate inhibitor for the treatment of COVID-19. However, major concern about such treatment method is its side effect inside the human body, considering some of Cytarabine side effect when used as a chemotherapeutic agent for Leukemia, further studies are needed to evaluate its biological significance within humans to justify its overall significance.
